# Orthobiologics in anterior cruciate ligament injuries

**DOI:** 10.1002/jeo2.70905

**Published:** 2026-09-12

**Authors:** Marc Daniel Bouchard, José Leonardo Rocha De Faria, Gustavo Vinagre, Douglas Mello Pavão, Nelson Merchan, Emmanouil Papakostas, Gilbert Moatshe, Pedro Bernáldez Domínguez, Theodorakys Marín Fermín, Ignacio Dallo, Ashraf Hantouly

**Affiliations:** ^1^ Division of Orthopedic Surgery, Department of Surgery McMaster University Hamilton Ontario Canada; ^2^ Knee Surgery Center, Orthobiologics Center National Institute of Traumatology and Orthopedics Rio de Janeiro Brazil; ^3^ Institute of Orthopedics and Traumatology, Hospital das Clínicas, Faculty of Medicine University of São Paulo (IOT‐HCFMUSP) São Paulo Brazil; ^4^ Department of Orthopaedic Surgery and Traumatology Unidade Local de Saúde do Médio Ave Porto Portugal; ^5^ Clínica Dr. Gustavo Vinagre Porto Portugal; ^6^ Santa Teresa Hospital Petropolis Rio de Janeiro Brazil; ^7^ Department of Orthopedic Surgery Massachusetts General Hospital Boston Massachusetts USA; ^8^ Harvard Combined Orthopaedic Surgery Boston Massachusetts USA; ^9^ Surgery Department Aspetar Orthopaedic and Sports Medicine Hospital Doha Qatar; ^10^ Oslo University Hospital Oslo Norway; ^11^ University of Oslo, Institute for Surgical Research Oslo Norway; ^12^ Norwegian School of Sports Sciences Oslo Sports Trauma Research Center Oslo Norway; ^13^ Departament of Orthopaedic Surgery and Sports Medicine, SportMe Medical Center Unit of Biological Therapies and Ultrasounds OrtobioSevilla Seville Spain; ^14^ Clínica Santa Sofía Caracas Venezuela; ^15^ Present address: Fowler Kennedy Sport Medicine Clinic University of Western Ontario London Ontario Canada

**Keywords:** anterior cruciate ligament, bone marrow aspirate concentrate, mesenchymal cells, orthobiologics, platelet‐rich plasma

## Abstract

Anterior cruciate ligament (ACL) injury remains a major challenge in sports medicine because it affects a young and active population and is associated with instability, secondary meniscal and chondral damage, delayed return to sport and post‐traumatic osteoarthritis. This narrative review provides an ACL‐specific overview of orthobiologics, with an emphasis on the biologic rationale, current clinical applications and the strength of the available evidence. The biologic limitations of the native ACL, particularly its poor intrinsic healing capacity in the intra‐articular environment, have spurred interest in strategies to improve graft ligamentization, tendon–bone healing, modulation of inflammation and preservation of native ligament tissue. Platelet‐rich plasma is the most widely investigated orthobiologic in ACL surgery; however, clinical findings remain inconsistent, largely because of substantial heterogeneity in preparation methods, leukocyte content, activation protocols and delivery sites. Bone marrow aspirate concentrate may provide a broader cellular and cytokine profile, but current studies suggest that any benefit is limited mainly to selective early imaging or functional recovery parameters, without consistent improvement in longer‐term patient‐reported outcomes. Other cell‐based approaches, including adipose‐ and synovium‐derived therapies, remain investigational and lack sufficient evidence to support routine clinical use. In contrast, scaffold‐based biologic repair, particularly bridge‐enhanced ACL repair, currently represents the most convincing example of successful biologic translation in this field, with prospective clinical studies demonstrating non‐inferior outcomes compared with autograft reconstruction in appropriately selected patients. The available literature remains difficult to interpret because of inconsistent product characterization, variable indications, heterogeneous surgical techniques, frequent reliance on surrogate imaging outcomes and underpowered clinical trials. Orthobiologics in ACL surgery should therefore be considered selective adjuncts rather than standard treatment. Future progress will depend on indication‐specific trial design, improved reporting of biologic composition and delivery methods and prioritization of clinically relevant outcomes such as graft failure, knee stability, return to sport and long‐term joint preservation.

**Level of Evidence:**

Level V, narrative review.

**Abbreviations**
ACL: anterior cruciate ligament
ACLR: anterior cruciate ligament reconstruction
BEAR: bridge‐enhanced anterior cruciate ligament repair
BMAC: bone marrow aspirate concentrate
MRI: magnetic resonance imaging
PROMs: patient‐reported outcome measures
PRP: platelet‐rich plasma

## INTRODUCTION

Anterior cruciate ligament (ACL) rupture remains one of the most important injuries in sports medicine because it affects a predominantly young and active population and carries implications for return to sport, recurrent instability, secondary meniscal damage and post‐traumatic osteoarthritis [4, 12, 19, 30]. Although contemporary ACL reconstruction (ACLR) generally provides reliable restoration of stability, it does not reproduce native ligament biology, requires graft harvest or allograft use and is still associated with graft failure, residual laxity, prolonged rehabilitation and incomplete return to preinjury performance in a substantial subset of patients [4, 6, 19, 30, 45].

These limitations have renewed interest in biologic augmentation. In ACL surgery, the central biologic goals are to improve graft ligamentization, accelerate tendon–bone integration, modulate the early inflammatory response, reduce tunnel widening and potentially facilitate repair of the native ligament in carefully selected tears [2, 11, 13, 18]. This topic is therefore not a generic discussion of orthobiologics in sports medicine. Rather, the relevant question for knee surgeons is which biologic adjuncts have a plausible mechanistic rationale in ACL injury, and which of these have translated into clinically meaningful benefits.

Earlier reviews often grouped together basic science, nonoperative injection studies, ACL repair and ACLR, making it difficult for readers to understand where the evidence is strongest and where it remains preliminary [11, 13, 18]. In addition, many reports inadequately describe the final biologic product, including platelet concentration, leukocyte content, activation method, harvest site, cell count and delivery location. This heterogeneity has limited meta‐analysis and has likely contributed to the disconnect between encouraging laboratory findings and inconsistent clinical outcomes [10, 11].

The purpose of this narrative review is therefore to provide an ACL‐specific synthesis of orthobiologics with an emphasis on clinically relevant decision‐making. The review is organized around ACL biology, the principal orthobiologic platforms used in ACL care, their application in reconstruction and repair, current limitations of the literature and practical priorities for future investigation.

### ACL biology and rationale for biologic augmentation

The ACL is composed predominantly of Type I collagen and fibroblast‐like cells arranged in a highly organized hierarchical structure. Its vascular supply is modest and regionally variable, and because it is an intra‐articular but extrasynovial structure, the biologic environment after rupture differs from that of extra‐articular ligaments [13, 29, 39, 56].

Unlike the medial collateral ligament, the ruptured ACL does not predictably heal through formation of a stable fibrin‐platelet provisional scaffold. Synovial fluid and intra‐articular plasmin activity interfere with clot persistence, while the gap between torn ligament ends and ongoing joint motion further disrupts the reparative environment [44, 56]. This biologic limitation explains why spontaneous healing of complete midsubstance tears is inconsistent and why traditional primary suture repair historically produced unacceptable failure rates [13].

In the setting of ACLR, the biologic challenge changes. The implanted graft undergoes necrosis, recellularization, revascularization, collagen remodeling and maturation, a process broadly referred to as ligamentization [39, 56]. Simultaneously, the graft must heal to bone within the femoral and tibial tunnels [2, 25]. Both processes are biologically slow and may be influenced by growth factor signaling, local inflammation, mechanical environment, graft type, fixation and rehabilitation progression [39].

**Figure 1:**
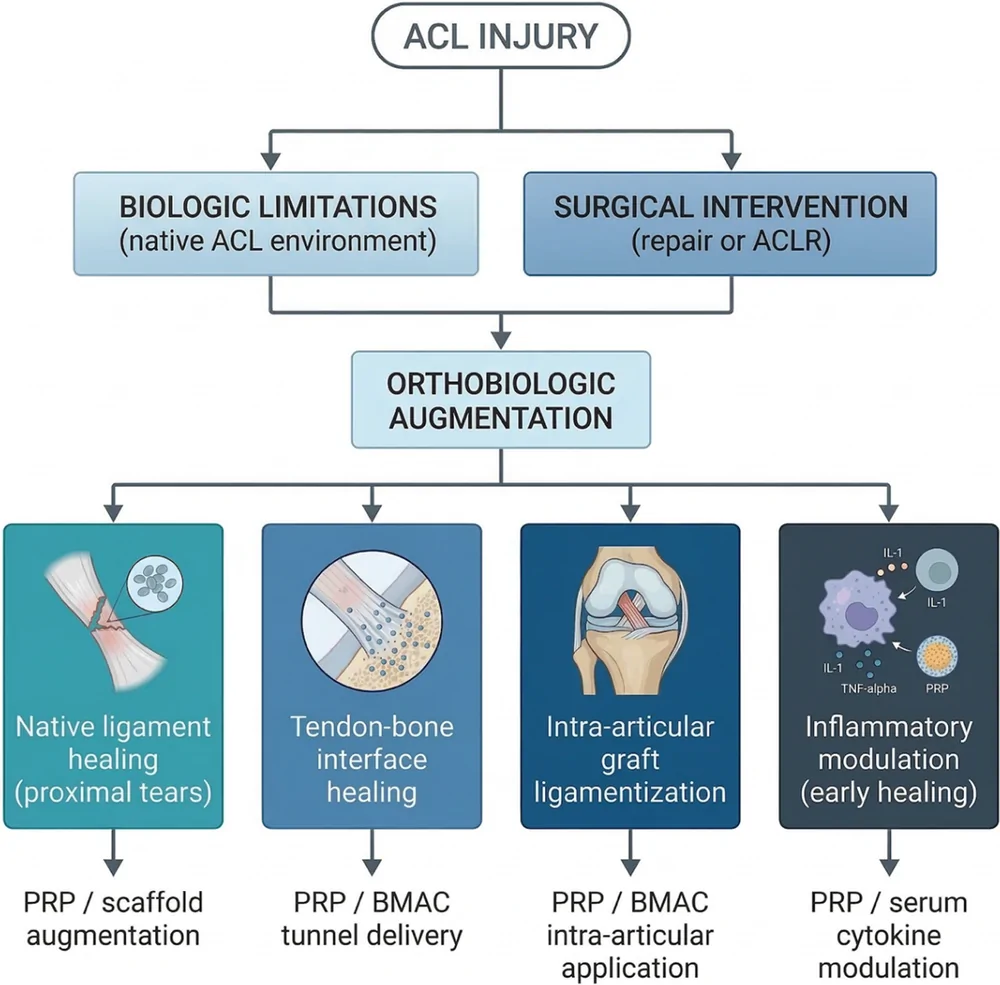
Conceptual framework for orthobiologic augmentation in ACL surgery. Schematic illustrating key biologic targets for augmentation, including native ligament healing, tendon–bone interface healing, graft ligamentization and modulation of the postoperative inflammatory environment. ACL, anterior cruciate ligament; ACLR, anterior cruciate ligament reconstruction; BMAC, bone marrow aspirate concentrate; PRP, platelet‐rich plasma.

Orthobiologics are attractive because they may modulate one or more of these steps. Platelet‐based products provide a concentrated milieu of cytokines and growth factors. Bone marrow aspirate concentrate (BMAC) adds mononuclear cells, progenitor populations and additional signaling molecules [11, 39]. Scaffold‐based technologies seek to retain the biologic payload at the injury site and provide a provisional matrix that the native ACL otherwise lacks [2, 11, 13, 18, 37]. These biologic targets within ACL healing are illustrated conceptually in Figure 1. Although the underlying rationale is scientifically compelling, the key question remains whether these strategies translate into clinically meaningful improvements in outcomes that matter to patients and surgeons.

### Orthobiologic platforms relevant to ACL surgery

**Figure 2:**
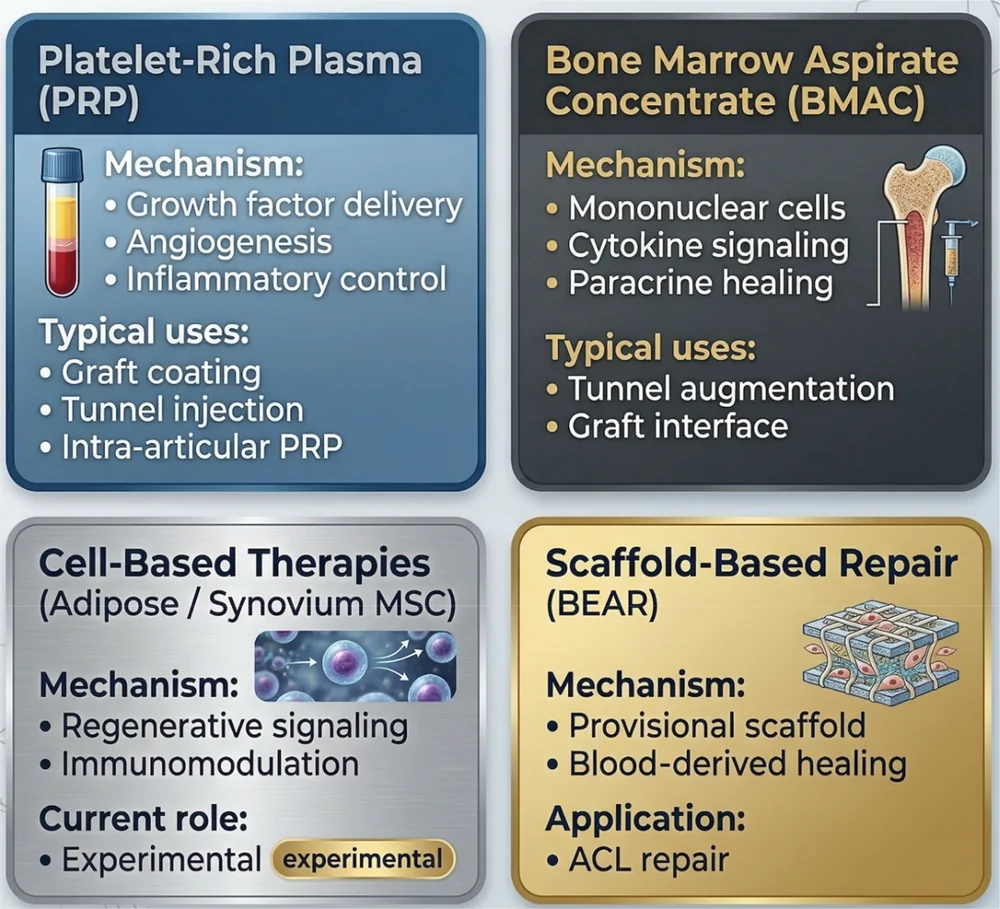
Orthobiologic platforms relevant to ACL injury and reconstruction. Overview of major orthobiologic platforms used in ACL surgery, including PRP, BMAC, cell‐based therapies and scaffold‐based repair strategies. ACL, anterior cruciate ligament; BEAR, bridge‐enhanced anterior cruciate ligament repair; BMAC, bone marrow aspirate concentrate; MSC, mesenchymal stem cells; PRP, platelet‐rich plasma.

The principal orthobiologic platforms currently investigated in ACL surgery, along with their biologic rationale, clinical applications and major limitations, are summarized in Table 1. Figure 2 provides a conceptual overview of these orthobiologic platforms and their mechanisms of action in ACL surgery.

**Table 1:** Orthobiologics platforms relevant to ACL surgery.

Product	Primary biologic rationale	Typical ACL application	Current limitation
PRP	High local concentration of platelet‐derived growth factors may modulate inflammation, angiogenesis and fibroblast activity	Graft coating, tunnel application, intra‐articular injection, repair augmentation	Marked heterogeneity of formulation and inconsistent long‐term clinical benefit
BMAC	Provides platelets, cytokines, mononuclear cells and rare mesenchymal progenitors that may support tendon–bone healing	Tunnel augmentation, graft interface delivery, combined augmentation constructs	Low‐quality and heterogeneous evidence; cell count often underreported
Adipose‐/synovium‐derived cell therapies	Potential paracrine support and multilineage differentiation	Experimental repair/regenerative strategies	Predominantly preclinical or early‐phase clinical evidence
Collagen scaffold/BEAR	Provides a provisional bridge and retains autologous blood at tear site to support native ACL healing	Primary ACL repair in selected proximal tears	Indication‐specific; not applicable to all tear patterns or chronic injuries
Autologous conditioned serum/other serum products	Anti‐inflammatory cytokine modulation	Adjunct to ACLR in select studies	Sparse ACL‐specific evidence

Abbreviations: ACL, anterior cruciate ligament; ACLR, anterior cruciate ligament reconstruction; BEAR, bridge‐enhanced anterior cruciate ligament repair; BMAC, bone marrow aspirate concentrate; PRP, platelet‐rich plasma.

#### Platelet‐rich plasma (PRP)

PRP is the most commonly studied orthobiologic in ACL surgery. It is produced from autologous peripheral blood and may be delivered as liquid PRP, gel or fibrin matrix. PRP contains platelet‐derived growth factor, transforming growth factor‐beta, vascular endothelial growth factor, insulin‐like growth factor‐1, fibroblast growth factor, epidermal growth factor, interleukins and other bioactive mediators that can influence angiogenesis, cell migration, collagen synthesis and inflammatory signaling [8, 11, 22, 46].

From an ACL perspective, PRP has been used at several targets: coating the graft before implantation, filling the femoral or tibial tunnels, wrapping the graft in PRP‐soaked matrices, injecting PRP intra‐articularly at the end of surgery and augmenting repair constructs [2, 3, 25]. However, PRP is not a single product. Leukocyte‐rich versus leukocyte‐poor preparations, platelet concentration, activation method and fibrin architecture all influence the release kinetics of growth factors and the potential inflammatory profile [8, 10, 17].

This lack of standardization is one of the major reasons the clinical literature has been difficult to interpret. A positive study using one PRP formulation and one delivery site cannot be assumed to apply to other preparations. For this reason, any ACL review that discusses PRP without defining the product risks overstating the evidence.

#### Bone marrow aspirate and BMAC

BMAC is typically harvested from the iliac crest, proximal tibia or other marrow source and concentrated intraoperatively [20, 28, 35]. It contains mesenchymal stromal cells in low absolute numbers, hematopoietic cells, endothelial progenitor cells, platelets, cytokines and growth factors such as platelet‐derived growth factors, tumor growth factor‐beta, vascular endothelial growth factor and bone morphogenetic proteins [20, 28, 35]. In ACL surgery, BMAC is generally used to enhance graft incorporation at the tendon–bone interface or to support repair biology [28, 35, 46].

The mechanistic appeal of BMAC is broader than that of PRP, but its clinical interpretation is complicated by the same heterogeneity problems. Cell yield varies by harvest site, aspiration technique, processing system and reporting method. In many studies, the actual mononuclear cell count or mesenchymal progenitor concentration is not reported, making comparisons across trials problematic [20, 28].

Importantly, BMAC should not be conflated with culture‐expanded stem cell therapy. Most commercially used BMAC preparations contain a very small fraction of mesenchymal progenitors, and their primary effect is likely paracrine rather than direct tissue replacement [28, 35].

#### Adipose‐ and synovium‐derived cell therapies

Adipose‐derived and synovium‐derived mesenchymal stromal cell strategies are attractive because these tissues may provide cells with proliferative, immunomodulatory and multilineage differentiation potential [33, 47]. In theory, these therapies could support ACL healing or improve the biologic environment of repair. In practice, however, their use in ACL surgery remains largely experimental [33, 47]. The majority of published work is preclinical, early‐phase or not directly specific to standard ACLR.

A key distinction for clinicians is that enthusiasm around ‘stem cells’ often exceeds the maturity of the ACL‐specific evidence. These products may eventually play a role in biologically augmented repair or revision settings, but they cannot currently be considered established adjuncts in routine ACL practice.

#### Scaffold‐based biologics and bridge‐enhanced anterior cruciate ligament repair (BEAR)

Scaffold‐based approaches attempt to solve a central biologic problem of ACL healing: the absence of a stable provisional bridge between torn ligament ends. The BEAR strategy combines a protein‐based extracellular matrix scaffold with autologous blood to create a biologically active bridge that supports healing of the native ACL [23, 40]. This concept is fundamentally different from simply injecting PRP or BMAC into a joint or tunnel because it provides both a biologic substrate and a physical healing environment.

Among orthobiologic approaches in ACL surgery, scaffold‐based repair currently has the strongest procedure‐specific clinical evidence. This is important because it shifts the discussion away from the broad question of whether orthobiologics work toward the more useful question of which biologic platform is best matched to which biologic problem.

### Preclinical evidence

Animal and laboratory studies generally support the biologic plausibility of orthobiologic augmentation in ACL surgery. In porcine and canine models, collagen‐PRP composites have improved histologic appearance, vascularity and some biomechanical properties of healing ACL tissue [27, 41, 42, 57]. PRP has also been associated with enhanced graft revascularization and reinnervation in reconstruction models [57].

However, preclinical findings must be interpreted carefully. Some studies demonstrated that PRP alone was insufficient to improve ACL healing in the intra‐articular environment, whereas PRP combined with collagen scaffolds generated better results [27, 41, 42]. These observations support the concept that retention and structural support may be as important as the biologic payload itself. In other words, a favorable laboratory signal does not imply that a simple injection strategy will succeed clinically.

This distinction likely explains part of the divergence between promising basic science and inconsistent human reconstruction trials. It also supports the growing interest in biologically active scaffolds for ACL preservation.

### Orthobiologics in ACLR

#### PRP during ACLR

PRP has been studied in ACLR more than any other biologic adjunct. Proposed benefits include accelerated graft maturation on magnetic resonance imaging (MRI), improved tendon–bone healing, reduced tunnel widening, lower postoperative pain and earlier recovery of stability [2, 3, 16, 38, 48, 50, 51]. Despite this strong rationale, the clinical literature remains mixed.

Early randomized and comparative studies showed variable results. Some reported more favorable graft maturation signals or lower pain scores in the early postoperative period, while others found no significant benefit in clinical scores, laxity or tunnel widening [3, 17, 50, 51]. Importantly, many of these studies were underpowered, used different grafts and fixation methods and delivered PRP at different targets.

Systematic reviews initially concluded that PRP did not provide consistent benefit in ACLR. For instance, Davey et al. found no clear advantage in graft healing, donor‐site morbidity, postoperative pain or functional outcomes across randomized trials [16]. More recent syntheses suggest a more nuanced view. Updated meta‐analytic data indicate that PRP may confer modest early improvements in knee stability or pain, particularly in selected formulations or delivery strategies, but these benefits have not translated into consistent superiority in long‐term patient‐reported outcome measures (PROMs), graft failure rates or return to sport [38, 48].

For the practicing surgeon, the most evidence‐based conclusion is not that PRP is ineffective, but rather that any benefit appears selective, formulation‐dependent and likely modest. This does not support universal routine PRP use during all primary ACLRs, especially when added cost and operative complexity are considered.

#### BMAC during ACLR

Compared with PRP, BMAC augmentation in ACLR is supported by a smaller but evolving body of evidence. Theoretically, BMAC may improve tendon–bone healing and graft maturation through a broader cellular and cytokine profile [28, 31, 34, 35, 43, 46]. Recent prospective studies and meta‐analyses, however, suggest that the clinical benefit remains uncertain [31, 34, 43].

A randomized prospective trial comparing control, PRP, and BMAC plus PRP found limited overall clinical benefit, with some early imaging and inflammatory differences but no clear superiority in standard postoperative outcome measures [34]. A recent systematic review and meta‐analysis of randomized controlled trials concluded that BMAC augmentation may improve aspects of graft recovery, but does not clearly improve early PROMs [43]. Likewise, a recent prospective comparative study evaluating BMAC with demineralized bone matrix and suture tape augmentation reported faster early range of motion and limb symmetry recovery, but similar 2‐year patient‐reported outcomes compared with non‐augmented ACLR [31].

These studies are important because they suggest that biologic augmentation may influence the pace of early recovery without necessarily altering the final outcome. That distinction matters clinically: Faster restoration of motion or strength can be useful, but unless it translates into lower failure rates, safer return to sport or improved long‐term function, it does not justify routine adoption on its own. A summary of the current clinical evidence for orthobiologic augmentation in ACLR is presented in Table 2.

**Table 2:** Clinical evidence for orthobiologic augmentation in ACL reconstruction.

Biologic	Key proposed mechanism	Representative clinical evidence	Main clinical findings
PRP	Growth factor delivery, angiogenesis, inflammatory modulation	Multiple RCTs and systematic reviews [3, 16, 38, 48, 50, 51]	Inconsistent benefit; possible early improvements in pain or MRI graft maturation, but no consistent improvement in PROMs, failure rates or return to sport
BMAC	Cellular and cytokine support for tendon–bone healing	Randomized and comparative studies [31, 34, 43]	May influence early graft recovery or rehabilitation metrics but no clear superiority in long‐term outcomes
Autologous conditioned serum	Anti‐inflammatory cytokine modulation	RCT evaluating intra‐articular conditioned serum [14]	Reduced tunnel widening and inflammatory markers, but limited replication
PRP + scaffolds	Growth factor delivery combined with biologic matrix retention	Early reconstruction and repair augmentation studies [2, 25]	Mechanistic promise but heterogeneous clinical data

Abbreviations: ACL, anterior cruciate ligament; BMAC, bone marrow aspirate concentrate; MRI, magnetic resonance imaging; PROMs, patient‐reported outcome measures; PRP, platelet‐rich plasma; RCT, randomized controlled trial.

#### Other serum‐based adjuncts

Autologous conditioned serum has also been explored in ACLR. For example, Darabos et al. reported reduced tunnel widening and lower synovial interleukin‐1 levels with intra‐articular conditioned serum after reconstruction [14]. Although intriguing, this strategy has not been widely replicated, and the available evidence is too sparse to support broad use. Nevertheless, it highlights an underexplored concept: not all biologic benefit in ACL surgery must come from growth factor stimulation; modulation of the inflammatory environment may be equally important.

### Orthobiologics in ACL repair and preservation

Historically, primary ACL repair fell out of favor because of high failure rates. Renewed interest has been driven by better patient selection, modern fixation methods and biologic augmentation. In this setting, orthobiologics may be more central to success than in standard reconstruction because they aim to support healing of the native ligament rather than merely accelerate graft incorporation [13, 25, 37].

#### BEAR

BEAR has become the leading biologically augmented repair strategy [23, 40]. The technique places an extracellular matrix scaffold between the torn ACL ends and saturates the scaffold with autologous blood, thereby recreating the provisional healing bridge missing in the native intra‐articular environment [23, 40]. In a prospective randomized trial, BEAR was found to be non‐inferior to autograft ACLR at 2 years for patient‐reported and functional outcomes in appropriately selected patients [40]. Medium‐term follow‐up from the first‐in‐human cohort has further supported the durability of this concept, with encouraging 6‐year outcomes [23].

The importance of BEAR extends beyond the procedure itself. It provides proof of principle that ACL preservation can succeed when the biologic and mechanical environment are jointly addressed. This is arguably the strongest clinical validation of orthobiologic principles currently available in ACL surgery. Major orthobiologic strategies currently investigated for ACL repair and preservation are summarized in Table 3.

**Table 3:** Orthobiologic strategies for ACL repair and preservation.

Strategy	Biologic concept	Typical indication	Representative evidence
BEAR Scaffold	Collagen scaffold saturated with autologous blood providing a provisional healing bridge	Acute proximal ACL tears suitable for repair	Randomized trial showing non‐inferiority to ACLR at 2 years [40]; 6‐year follow‐up demonstrating durable outcomes [23]
Biologically Augmented Primary Repair	Repair with marrow stimulation, biologic scaffold or biologic injection	Selected proximal tears or incomplete injuries	Clinical series reporting favorable outcomes in selected patients [1, 25]
Injection‐based Biologic Therapy	PRP/BMAC injection to stimulate ligament healing	Selected partial tears or low‐demand patients	Registry and early‐phase studies with limited comparative evidence [54]
Cell‐based Regenerative Strategies	Adipose or synovial mesenchymal stromal cell delivery	Experimental repair or regenerative strategies	Predominantly preclinical or early‐phase research [33, 47]

Abbreviations: ACL, anterior cruciate ligament; ACLR, anterior cruciate ligament reconstruction; BEAR, bridge‐enhanced anterior cruciate ligament repair; BMAC, bone marrow aspirate concentrate; PRP, platelet‐rich plasma.

### Non‐scaffold ACL preservation strategies

Contemporary ACL preservation also includes non‐scaffold approaches whose principal therapeutic role is mechanical protection of the repaired native ligament, including dynamic intraligamentary stabilization (DIS/Ligamys), suture‐tape/InternalBrace augmentation and approaches described as retro‐ligamentary filling. Dynamic intraligamentary stabilization provides load‐sharing stabilization during ligament healing, and a systematic review of five randomized controlled trials found broadly comparable short‐ to mid‐term patient‐reported outcomes, failure and revision rates to hamstring ACLR, although ACLR demonstrated greater objective stability at selected time points [1]. For suture‐augmented primary repair, a recent comparative meta‐analysis found no significant differences versus ACLR in re‐rupture rates (11.5% vs. 8.4%), patient‐reported outcomes or return to sport, although a small difference in side‐to‐side laxity favoured reconstruction [7]. More broadly, a 2026 meta‐analysis found higher failure rates and greater residual laxity after arthroscopic ACL repair than ACLR, particularly when repair was performed more than 3 weeks after injury, underscoring the importance of appropriate timing and patient and tear selection [54]. These mechanically augmented preservation strategies are conceptually distinct from BEAR, which couples ligament repair with an extracellular‐matrix scaffold and autologous blood to recreate a biologically active provisional bridge at the rupture site [23, 40].

Importantly, these ACL preservation strategies should not themselves be considered orthobiologic therapies. Rather, techniques such as dynamic intraligamentary stabilization and suture‐tape augmentation primarily provide mechanical protection of the healing ligament while preserving native ACL tissue. This distinction is relevant within the context of orthobiologics, as preservation of the native ligament may provide a biologically active substrate upon which adjunctive therapies can act.

#### Repair of partial or proximal tears with biologic augmentation

Biologically augmented repair has also been described for partial tears and proximal avulsion‐type injuries, including techniques using marrow stimulation, hyaluronic scaffolds and adjunctive biologics [25, 26]. Gobbi and Whyte reported favorable long‐term results after repair of incomplete tears with biologic healing augmentation in selected patients [26]. These findings suggest that orthobiologics may be particularly relevant in tear patterns with preserved tissue quality and repairable anatomy.

However, these indications are narrow. Chronic tears, midsubstance ruptures with poor tissue quality, and cases with substantial retraction remain poor candidates for repair‐based biologic approaches. This reinforces the need to match the orthobiologic strategy to the underlying pathology rather than presenting all ACL injuries as equivalent candidates for augmentation.

### Nonoperative and injection‐based strategies

There has been growing public and commercial interest in injection‐based orthobiologics for ACL injury, particularly combinations of marrow concentrate and platelet products. Registry and early‐phase studies suggest these approaches may be safe and potentially beneficial in selected partial tears or low‐demand patients [9]. However, the evidence remains limited, non‐comparative and highly susceptible to selection bias.

**Figure 3:**
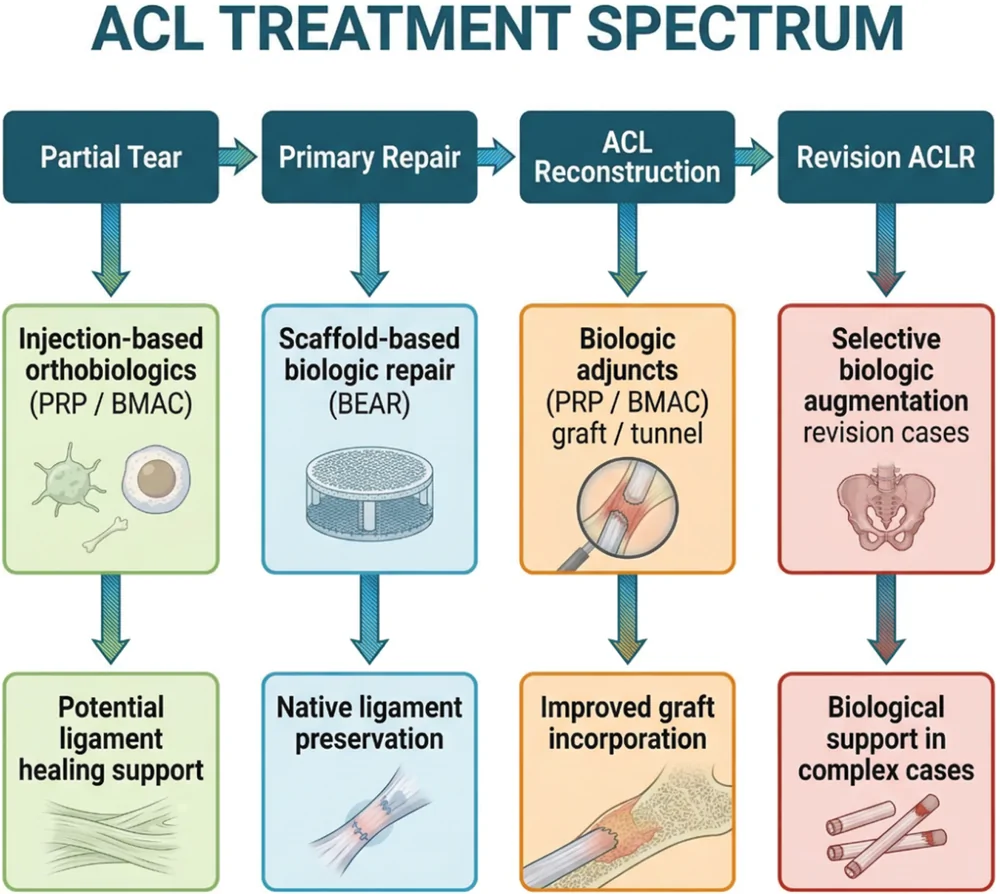
Clinical application map of biologic augmentation across the ACL treatment spectrum. Illustration demonstrating where orthobiologics may be considered across different ACL treatment strategies, including partial tears, primary repair, standard ACLR and revision reconstruction. ACL, anterior cruciate ligament; ACLR, anterior cruciate ligament reconstruction; BEAR, bridge‐enhanced anterior cruciate ligament repair; BMAC, bone marrow aspirate concentrate; PRP, platelet‐rich plasma.

These strategies should therefore be interpreted cautiously. They may have a future role in carefully defined indications, especially partial tears with preserved continuity, but they should not be conflated with validated alternatives to ACLR in active patients with complete ruptures and functional instability. The potential clinical applications of orthobiologics across the ACL treatment spectrum are illustrated in Figure 3.

### Why the clinical literature remains inconclusive

A major reason orthobiologic studies in ACL surgery have been difficult to synthesize is that many trials combine biologically distinct problems under a single label. An intervention aimed at improving tendon–bone healing in ACLR is fundamentally different from one intended to support native ligament healing in ACL repair. Similarly, intra‐articular PRP injection is different from tunnel placement, scaffold impregnation or graft wrapping. Pooling these applications risks obscuring signal with noise.

Product characterization is another major limitation. Many PRP studies fail to report platelet count, leukocyte status, activation method and final volume [8, 20]. BMAC studies often omit aspiration details, nucleated cell count, processing system and final injectate composition [28, 35]. Without this information, replication and comparison are difficult.

Outcome selection is also problematic. Imaging‐based graft ‘maturity’ or tunnel changes may be biologically interesting but are surrogate endpoints. Unless they correlate with laxity, retear risk, return to sport or patient‐reported function, their clinical value remains uncertain. Several orthobiologic studies report MRI differences without demonstrating superior long‐term clinical outcomes [16, 38, 48], which may partly explain why surgeon enthusiasm and evidence‐based consensus have diverged.

Finally, many studies are underpowered, single‐center and involve multiple technical confounders such as graft type, fixation, concomitant procedures, rehabilitation variation and return‐to‐sport criteria. Future trials need to isolate the biologic question more rigorously.

Key reporting priorities that may improve future orthobiologic trials in ACL surgery are outlined in Table 4.

**Table 4:** Proposed reporting priorities for future ACL orthobiologic studies.

Domain	What should be reported	Why it matters
Product Characterization	Platelet count, leukocyte status, activation method, final volume, scaffold carrier, harvest site, cell count/mononuclear fraction	Allows reproducibility and meaningful comparison across studies
Indication	ACLR versus repair versus partial tear/nonoperative injection	These are biologically distinct use cases and should not be pooled indiscriminately
Delivery Details	Location of application (graft, tunnel, intra‐articular, scaffold), timing, number of doses	Therapeutic effect may depend on where and how the product is delivered
Clinical Outcomes	PROMs, laxity, graft failure, return to sport, reoperation, adverse events, cost	Improves external validity and clinical relevance
Imaging	Standardized MRI protocol and correlation with clinical outcomes	Prevents overinterpretation of surrogate biologic endpoints

Abbreviations: ACL, anterior cruciate ligament; ACLR, anterior cruciate ligament reconstruction; MRI, magnetic resonance imaging; PROMs, patient‐reported outcome measures.

### Practical clinical implications

At present, orthobiologics in ACL surgery should be viewed as adjuncts rather than replacements for sound surgical technique, graft selection, tunnel positioning, fixation and structured rehabilitation. For most surgeons performing standard primary ACLR, the available evidence does not support routine universal use of PRP or BMAC solely to improve final patient‐reported outcomes.

That said, selective application may be reasonable. Examples include revision settings, cases with biologic concern at the tendon–bone interface, research protocols or repair‐preservation strategies in carefully chosen proximal tears. Even in these situations, the rationale for use should be explicit and expectations should remain realistic.

The most clinically compelling biologic signal in ACL surgery currently comes from scaffold‐based repair, particularly BEAR, rather than isolated injectable augmentation [23, 40, 49]. This distinction is important because it suggests that successful orthobiologic strategies may require both biologic activity and structural retention at the healing site.

### Regulatory, safety and economic considerations

Regulatory considerations are important when translating orthobiologic therapies into clinical practice. In the United States, human cells, tissues and cellular and tissue‐based products (HCT/Ps) are assessed under 21 CFR Part 1271, including the criteria of minimal manipulation and homologous use [53]. Products that do not satisfy the applicable criteria may be subject to additional regulatory requirements and premarket review. Importantly, this framework should not be applied indiscriminately to all orthobiologics; the U.S. Food and Drug Administration (FDA) specifically considers autologous PRP a blood product rather than an HCT/P [52]. In the European Union, cell‐ and tissue‐based products may meet advanced therapy medicinal product (ATMP) definitions when cells or tissues are substantially manipulated or are intended for a different essential function in the recipient, with classification remaining product‐ and intended‐use specific [15, 21].

Safety profiles also vary across contemporary ACL preservation strategies. In the pivotal BEAR trial, reinjury requiring a second ipsilateral ACL procedure occurred in 14% of BEAR patients compared with 6% following autograft ACLR, although this difference was not statistically significant; preliminary post‐market data subsequently reported no retears or instability but postoperative arthrofibrosis in 5% [40, 49]. For dynamic intraligamentary stabilisation (DIS), a randomised trial reported clinical failure requiring revision in 16.3% compared with 12.5% following ACLR, while pooled comparative evidence identified a significantly higher rate of implant removal with DIS [1, 7]. Suture‐augmented primary ACL repair with an InternalBrace demonstrated a pooled rerupture rate of 10.6% and a non‐rerupture reoperation rate of 3.2%, predominantly related to hardware removal [54].

Autologous injectable orthobiologics have not demonstrated a prominent ACL‐specific safety signal, although adverse‐event reporting remains heterogeneous. PRP augmentation was not associated with increased complications or additional surgery in an ACLR comparative cohort [16, 36, 38, 48], while a meta‐analysis of randomised BMAC studies found complication rates comparable with controls [43]. These findings should not be extrapolated to substantially manipulated, culture‐expanded or allogeneic cellular products, which have distinct regulatory and safety considerations [21, 53, 55].

ACL‐specific cost‐effectiveness evidence remains limited. A 3‐year German cost‐utility model suggested that DIS produced modestly greater quality‐adjusted life‐years than early ACLR at higher cost, with an incremental cost‐effectiveness ratio of approximately €9093 per quality‐adjusted life‐year [5]. However, this analysis relied partly on assumptions and older cost inputs, and robust ACL‐specific economic evidence establishing the cost‐effectiveness of BEAR, InternalBrace, PRP or BMAC remains insufficient. Cost should therefore be considered alongside incremental clinical benefit, complications, reoperation and resource utilisation rather than as a stand‐alone argument for or against orthobiologic adoption.

### Future directions

**Figure 4:**
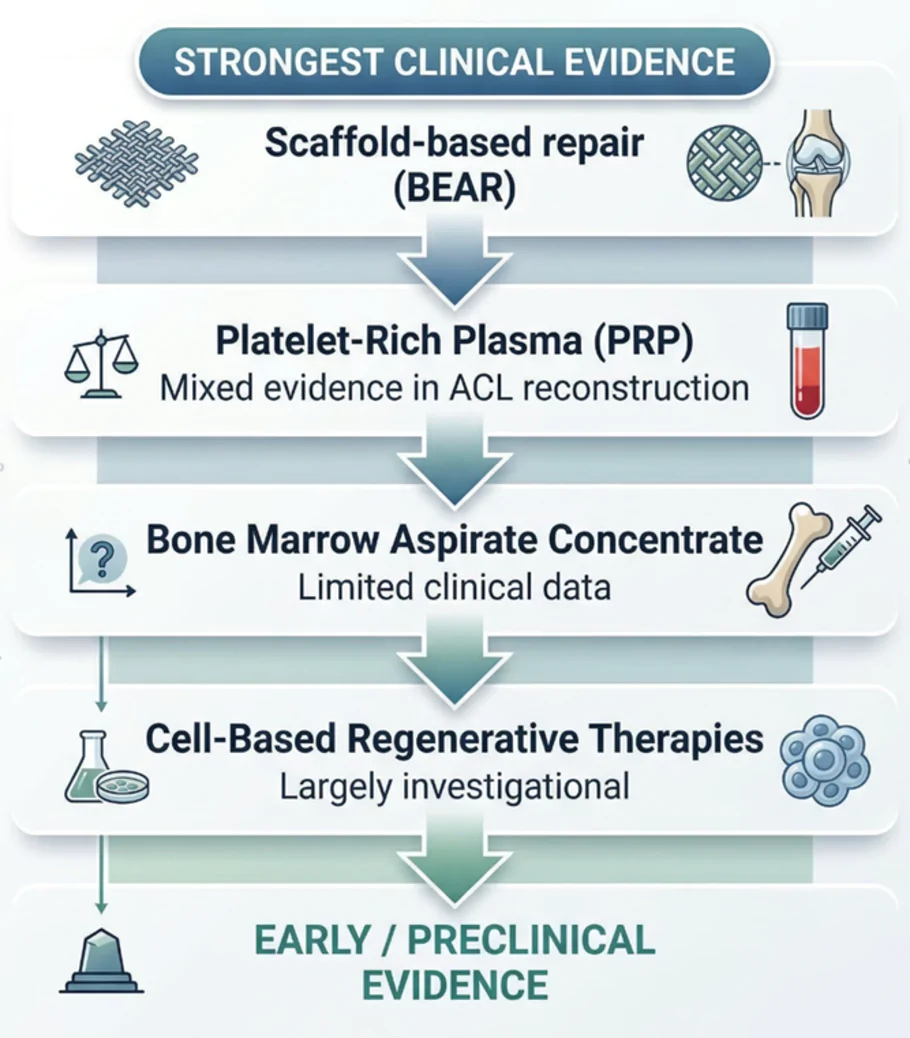
Evidence strength pyramid for orthobiologics in ACL surgery. Conceptual hierarchy illustrating the relative maturity of clinical evidence supporting scaffold‐based repair, PRP, BMAC and emerging cell‐based regenerative therapies. ACL, anterior cruciate ligament; BEAR, bridge‐enhanced anterior cruciate ligament repair; BMAC, bone marrow aspirate concentrate; PRP, platelet‐rich plasma.

Figure 4 illustrates the relative maturity of clinical evidence across currently investigated orthobiologic strategies in ACL surgery.

**Figure 5:**
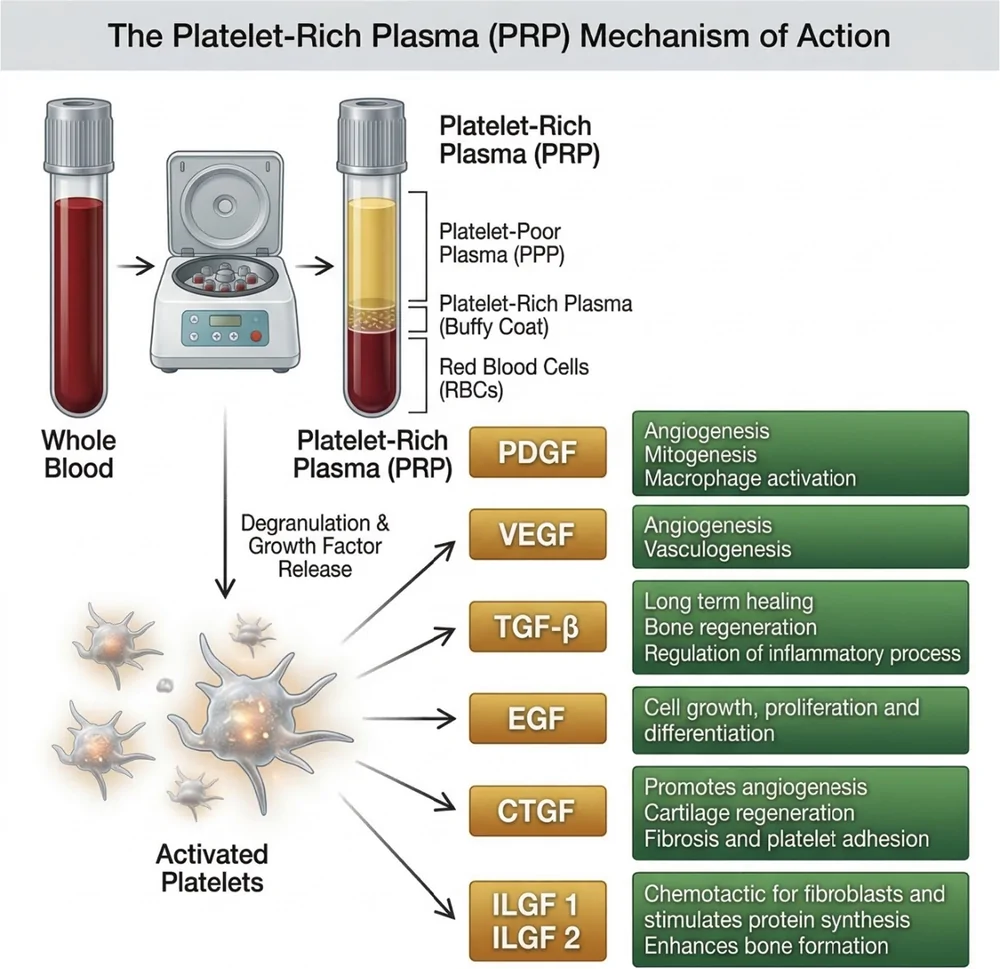
Mechanisms of action of platelet‐rich plasma in ACL healing. Schematic illustrating the proposed biologic mechanisms of PRP in ACL surgery. Following platelet activation, PRP releases a range of growth factors and cytokines, including PDGF, TGF‐β, VEGF and IGF‐1. These mediators may promote fibroblast proliferation, collagen synthesis, angiogenesis and modulation of the inflammatory response, contributing to graft ligamentization and tendon–bone interface healing. ACL, anterior cruciate ligament; CTGF, connective tissue growth factor; EGF, epidermal growth factor; IGF‐1, insulin‐like growth factor‐1; IGF‐2, insulin‐like growth factor‐2; PDGF, platelet‐derived growth factor; PRP, platelet‐rich plasma; TGF‐β, transforming growth factor–beta; VEGF, vascular endothelial growth factor.

In addition to differences in clinical evidence, important variability exists in the biologic composition, mechanism of action and delivery strategies of commonly used orthobiologics. PRP, the most widely studied biologic adjunct, demonstrates complex growth factor signaling pathways that may influence angiogenesis, collagen synthesis and inflammatory modulation (Figure 5).

**Figure 6:**
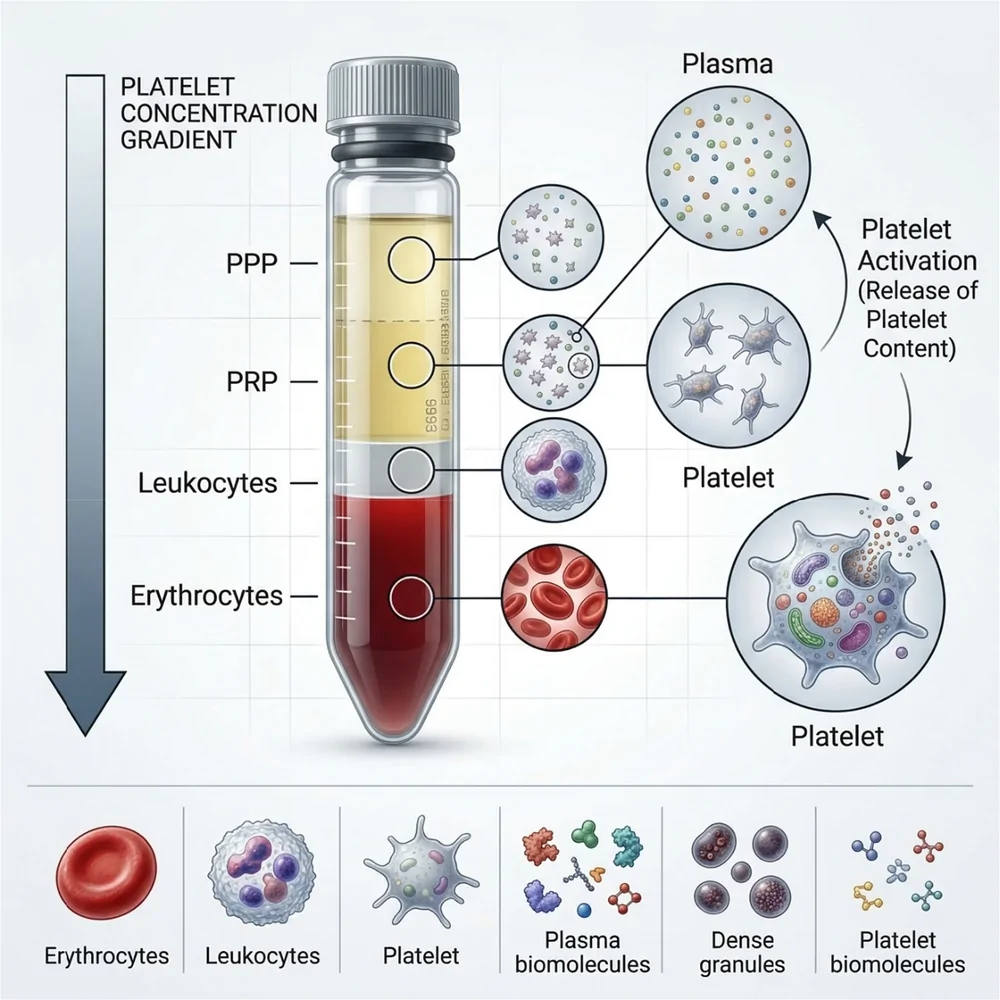
Platelet‐rich plasma composition and cellular gradient following centrifugation. Schematic illustrating the principal components of whole blood after centrifugation and the formation of PRP. The figure demonstrates the relative distribution of erythrocytes, leukocytes, PPP and PRP within the centrifuged sample, as well as the cellular and molecular elements released following platelet activation, including dense granules and platelet biomolecules. PPP, platelet‐poor plasma; PRP, platelet‐rich plasma.

The final composition of PRP is highly dependent on preparation technique, with centrifugation resulting in distinct cellular layers including platelet‐poor plasma, PRP, leukocytes and erythrocytes, which may influence biologic activity and clinical effect (Figure 6).

**Figure 7:**
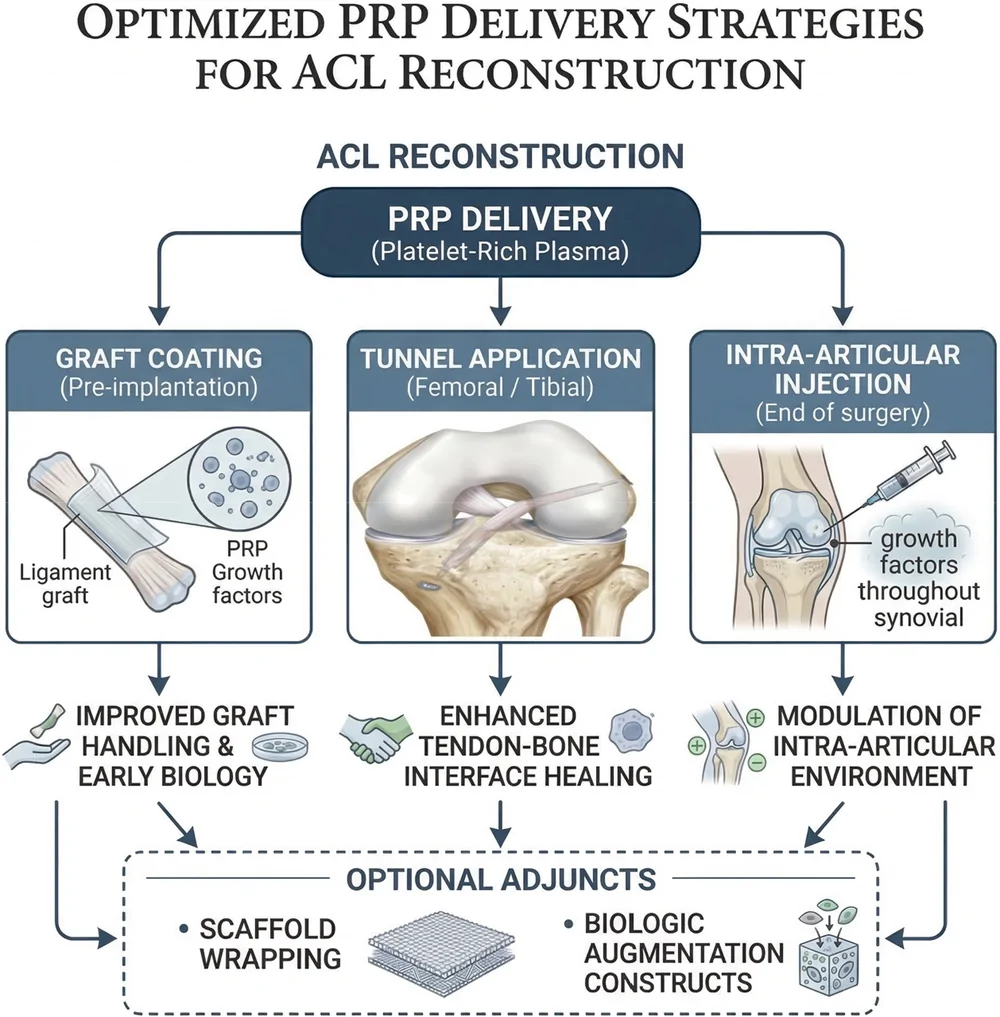
Clinical application sites of PRP in ACLR. Illustration demonstrating the common delivery strategies of PRP in ACLR. PRP may be applied to the graft prior to implantation, delivered within the femoral or tibial tunnels to enhance tendon–bone healing or injected intra‐articularly at the conclusion of surgery. Additional strategies include scaffold wrapping or incorporation into biologic augmentation constructs. The biologic effect of PRP may depend on the location and method of delivery. ACL, anterior cruciate ligament; ACLR, anterior cruciate ligament reconstruction; PRP, platelet‐rich plasma.

In addition, the clinical efficacy of PRP may depend on the site and method of delivery, including graft coating, tunnel application, intra‐articular injection or incorporation into scaffold‐based constructs, as illustrated in Figure 7.

Several priorities should shape the next phase of ACL orthobiologic research. First, product reporting must improve. PRP and BMAC studies should consistently define the final product and delivery protocol. Without this, even high‐quality randomized trials remain difficult to interpret. This emphasis is consistent with the European Society of Sports Traumatology, Knee Surgery and Arthroscopy (ESSKA)‐Orthobiologics Initiative (ORBIT) consensus programme on injectable orthobiologics, which has addressed blood‐derived products and, more recently, cell‐based therapies, with particular attention to indications, product preparation and characterisation and administration protocols [24, 32]. Although these consensus recommendations were developed for the nonoperative treatment of knee osteoarthritis rather than ACL injury, their emphasis on standardised terminology, product characterisation, patient selection and defined treatment protocols provides a relevant framework for the responsible clinical translation and investigation of orthobiologics in ACL surgery [24, 32]. Second, indication‐specific trials are needed. ACLR, ACL repair, partial tears, revision surgery and nonoperative injection strategies should not be grouped together indiscriminately. Third, outcomes should extend beyond early MRI findings. Future studies should include graft failure, instrumented laxity, return‐to‐sport timing and level, reoperation, complications and cost‐effectiveness. Fourth, combination strategies deserve attention. The future of biologic augmentation may lie less in single‐agent injection and more in systems that combine scaffold support, controlled biologic release and appropriate mechanical stabilization. Finally, patient selection may prove decisive. Biologic augmentation is unlikely to provide the same value in all ACL injuries. Proximal tears, pediatric or adolescent preservation candidates, revision settings and biologically compromised interfaces may represent the most rational early targets.

## CONCLUSIONS

Orthobiologics have become an increasingly important part of the scientific conversation surrounding ACL injury, reconstruction and repair. However, the literature should be interpreted through an ACL‐specific lens rather than as a general discussion of regenerative medicine. PRP remains the most studied orthobiologic in ACLR, but its clinical benefits appear inconsistent and generally modest. BMAC is promising but remains insufficiently defined and incompletely validated. Cell‐based therapies from adipose and synovial sources are investigational. In contrast, scaffold‐based biologic repair strategies, especially BEAR, have provided the strongest clinical support for orthobiologic principles in ACL preservation. The field is therefore promising, but not yet mature. The next advances will depend not simply on adding more biologics, but on aligning the right biologic platform with the right ACL pathology, standardizing product characterization and focusing on outcomes that truly matter to patients and surgeons.

## AUTHOR CONTRIBUTIONS

Gustavo Vinagre, José Leonardo Rocha de Faria, Ignacio Dallo and Ashraf Hantouly contributed to the conception and design of the review. Marc Daniel Bouchard, José Leonardo Rocha de Faria, Douglas Mello Pavão, Nelson Merchan and Ashraf Hantouly contributed to the literature review and drafting of the manuscript. Douglas Mello Pavão, Emmanouil Papakostas, Gilbert Moatshe, Pedro Bernáldez Domínguez, Theodorakys Marín Fermín, Ignacio Dallo and Ashraf Hantouly critically revised the manuscript and contributed to the interpretation of the available evidence. Ashraf Hantouly supervised and coordinated the project. All authors contributed to drafting the final manuscript and approved the final version.

## CONFLICT OF INTEREST STATEMENT

The author, Theodorakys Marín Fermín, is an Associate Editor for the Journal of Experimental Orthopaedics. Given his role, he had no involvement in the peer review of this article and no access to its information. Full responsibility for the editorial process for this article was delegated to another journal editor. The remaining authors declare no conflict of interest.

## ETHICS STATEMENT

The authors have nothing to report.

## Data Availability

Data sharing is not applicable to this article as no datasets were generated or analysed during the current study.
